# Adolescence is the starting point of sex-dichotomous *COMT* genetic effects

**DOI:** 10.1038/tp.2017.109

**Published:** 2017-05-30

**Authors:** S Sannino, M C Padula, F Managò, M Schaer, M Schneider, M Armando, E Scariati, F Sloan-Bena, M Mereu, M Pontillo, S Vicari, G Contarini, C Chiabrera, M Pagani, A Gozzi, S Eliez, F Papaleo

**Affiliations:** 1 Department of Neuroscience and Brain Technologies, Istituto Italiano di Tecnologia, Genoa, Italy; 2 Office Médico-Pédagogique, Department of Psychiatry, University of Geneva, Geneva, Switzerland; 3 Department of Neuroscience, Bambino Gesù Children’s Hospital, Piazza Sant’Onofrio, Rome, Italy; 4 Service of Genetic Medicine, University Geneva Hospitals, Geneva, Switzerland; 5 Dipartimento di Scienze del Farmaco, Universita’ degli Studi di Padova, Padova, Italy; 6 Functional Neuroimaging Laboratory, Center for Neuroscience and Cognitive Systems, Istituto Italiano di Tecnologia, Rovereto, Italy; 7 Center for Mind and Brain Sciences, University of Trento, Rovereto, Italy

## Abstract

The catechol-*o*-methyltransferase (*COMT*) genetic variations produce pleiotropic behavioral/neuroanatomical effects. Some of these effects may vary among sexes. However, the developmental trajectories of *COMT*-by-sex interactions are unclear. Here we found that extreme COMT reduction, in both humans (22q11.2 deletion syndrome COMT Met) and mice (*COMT*−/−), was associated to cortical thinning only after puberty and only in females. Molecular biomarkers, such as tyrosine hydroxylase, Akt and neuronal/cellular counting, confirmed that *COMT*-by-sex divergent effects started to appear at the cortical level during puberty. These biochemical differences were absent in infancy. Finally, developmental cognitive assessment in 22q11DS and *COMT* knockout mice established that *COMT*-by-sex-dichotomous effects in executive functions were already apparent in adolescence. These findings uncover that genetic variations severely reducing COMT result in detrimental cortical and cognitive development selectively in females after their sexual maturity. This highlights the importance of taking into account the combined effect of genetics, sex and developmental stage.

## Introduction

The catechol-*o*-methyltransferase (*COMT*) is a gene located in band q11.2 of chromosome 22,^1^ which encodes for the COMT enzyme, involved in the methylation of catechol molecules including dopamine, norepinephrine and catechol estrogens.^2^ In particular, COMT has a pivotal role in the degradation of dopamine in the prefrontal cortex (PFC).^3, 4, 5^ The Val/Met common and extensively studied single nucleotide polymorphism, hereafter referred to as ValMet, has been consistently reported to substantially change the stability and activity of COMT: the Met form leads to 40% lower protein levels and reduced enzymatic activity compared with the Val variant.^5^

Functional genetic variations in the *COMT* gene modulate multiple spheres of mammalian behavior with similar findings from adult humans and mice.^6, 7, 8, 9, 10, 11, 12^ However, if and how the effects of *COMT* genetic polymorphisms might change during the course of development is still unclear, with mixed and inconsistent findings. For example, two studies reported that MetMet homozygote children perform better than ValVal in PFC-dependent cognitive tests.^13, 14^ Conversely, other studies showed an advantage of ValVal or ValMet on PFC-dependent cognitive functions.^15, 16^ Moreover, including a cross-sectional and longitudinal study on a sample of healthy participants of 6–20 years old, the ValVal advantage observed in children was completely reverted in adolescence.^15^ Similarly, brain morphology investigations reported that 10-year-old ValVal children have reduced cortical thickness compared with MetMet subjects.^17^ This effect was completely reverted by the age of 20. The heterogeneity in these findings might indicate a different impact of *COMT* genetics throughout brain maturation, even if also other factors such as sample size, age distribution, experimental design and the effect of polymorphisms in other genes might have an influence. Indeed, COMT activity increases from childhood to adulthood;^18^ dopamine concentration/turnover in the PFC changes during lifetime, with a peak in adolescence;^19, 20, 21, 22^ the PFC morphology has a prolonged postnatal maturation.^23, 24, 25, 26^

Human and mouse studies have shown that behavior, brain morphology and vulnerability to psychiatric disorders modulated by *COMT* genetic variations are sex-dependent.^9, 27, 28, 29, 30, 31^ Moreover, estrogens inhibit COMT expression and enhance cortical dopamine, and COMT activity is lower in females than in males.^5, 29, 32, 33^ Only one study in healthy children from 8 to 10 years old suggested that the *COMT* ValMet polymorphism might impact cognitive function changes in relation to the pubertal status, but selectively in boys.^13^ Despite this, little is known about how COMT genetics might impact brain and behavioral maturational trajectories depending on the sex of the subjects.

Patients with 22q11.2 deletion syndrome (22q11DS) carry a hemizygous deletion of 1.5 (atypical form) or 3 (most frequent) megabases in the 22q11.2 locus, which include approximately 35 to 60 genes.^34^ 22q11DS individuals have only one copy of the *COMT* gene and are supposedly exposed to abnormal cortical dopamine levels throughout brain maturation.^10, 12, 35^ Furthermore, 22q11DS Met carriers have even lower COMT levels.^36^ 22q11DS is characterized by developmental cognitive impairments, high prevalence of attention deficit hyperactivity disorder (≈37%) from childhood and psychotic symptoms (≈30–40%) from adolescence/early adulthood.^37, 38, 39, 40^ In parallel, progressive brain morphological alterations are present, including greater gray matter loss in the parietal and occipital lobes with a relative preservation of the frontal lobe during childhood,^41, 42, 43, 44, 45, 46, 47^ and widespread alterations mainly involving temporal and frontal brain regions in adulthood.^46, 47^ Some studies suggested that the *COMT* ValMet variant might modulate brain and cognitive development,^48, 49, 50, 51, 52^ as well as risk for psychosis^35, 53^ in individuals with 22q11DS. However, a number of studies failed to detect any significant association of the ValMet variation with behavioral, neurocognitive, psychiatric and neural abnormalities.^36, 39, 51, 54, 55^ Thus, findings have been inconsistent and controversial.

The aim of this study was to clarify the developmental trajectories of *COMT*-by-sex interactions in the context of excessive changes of COMT activity. In particular, we conducted a translational study in patients affected by 22q11DS and COMT knockout mice. We first examined the influence of *COMT* ValMet polymorphism on cortical thickness in 22q11DS patients at different stages of sexual maturation, namely before and after puberty onset. Indeed, puberty has been indicated as a turning point in cortical development,^56, 57, 58^ in maturation of higher order cognitive functions^59^ and in the appearance of psychiatric disorders.^60^ Next, to dissect the effects of selective and drastic reductions in COMT, we took advantage of *COMT* knockout mice. These mutant mice allow to overcome the complexity of human polymorphisms, genetic and clinical heterogeneity and the potential confounding effects of other hemideleted genes in 22q11DS and other uncontrollable gene–gene and gene–environment interactions. We conducted a human-analogous magnetic resonance imaging (MRI) assessment comparing *COMT* null mutant −/− mice (an extreme situation approximating 22q11DS Met subjects) with their wild-type (+/+) and heterozygous (+/−) counterparts. Our results demonstrate that *COMT* genetics influence post-pubertal cortical brain anatomy in a sex-dependent manner in mice, consistent with what we found in 22q11DS. To strengthen our findings, we obtained molecular confirmation of altered developmental trajectories brought by *COMT*-by-sex interaction by measuring two key molecules implicated in dopamine pathways (TH and Akt) and neuronal/cellular density in the PFC of *COMT* male and female knockout mice at different developmental stages (that is, pre-pubertal, pubertal and post-pubertal). Finally, the repercussion of *COMT* genetics on mice and 22q11DS cognitive functions was investigated. At both neuroanatomical and behavioral levels, the interaction between *COMT* genetics and the sex of the subject appeared from puberty onwards.

## Materials and methods

### Human studies

22q11DS cohorts: 109 patients with 22q11DS were recruited and assessed (MRI, IQ, Stroop, CPT, digit span tests) in the context of the Geneva 22q11DS study,^38^ while other 83 patients with 22q11DS were recruited and assessed (Wisconsin Card Sorting task, IQ, Stroop, digit span tests) by the Bambino Gesù Hospital in Rome. The presence of the 22q11.2 microdeletion was confirmed by quantitative fluorescent polymerase chain reaction and hemizygosity for the *COMT* Met or the *COMT* Val allele was determined by PCR using a tetra-primer amplification refractory mutation system.^61^ In addition, sexual maturity was assessed using a self-questionnaire (Tanner maturational scale:^62, 63^ patients were classified at pre-pubertal stage (Tanner stage=1) or pubertal/post-pubertal stage (Tanner stage ⩾2)). For simplicity, we defined ‘post-pubertal’ all the subjects at a Tanner stage ⩾2. Eighteen subjects had both a data point in pre- and in post-puberty. Semi-structured interviews were used to assess the psychiatric diagnosis of the patients.^64, 65, 66^ See Supplementary Results for details on psychiatric diagnosis. Written informed consent was received from all the participants, and from their parents for participants younger than 18 years old, under protocols approved by the Institutional Review Board of Geneva University School of Medicine and Bambino Gesù Hospital.

### Structural MRI in humans

Structural MRI scans were acquired in the Geneva cohort only using either a Philips 1.5 Tesla Intera scanner or a Siemens Trio 3 Tesla scanner at the Center of Biomedical Imaging in Geneva (Supplementary Table 1 for groups assessed with each machine). Sequence parameters for the Philips 1.5 Tesla scanner were: TR=35 ms, TE=6 ms, flip angle=45°, NEX=1, matrix=256 × 192, field of view=24 cm^2^, slice thickness=1.5 mm, 124 slices; parameters for the Trio 3 Tesla scanner were: TR=2500 ms, TE=3 ms, flip angle=8°, acquisition matrix=224 × 256, field of view=220 mm, slice thickness=1.1 mm, 192 slices. Excellent cross-scanner vertex-wise consistency in cortical thickness estimates was previously confirmed using 20 participants who underwent cerebral MRI acquisitions with the two scanners on the same day.^67^ Structural images were processed using the software FreeSurfer version 5.1 to reconstruct accurate cortical surface models. Fully automated pre-processing included resampling into cubic voxels, intensity normalization and skull stripping. Three-dimensional surface reconstruction was used to determine the border between white and gray matter (white surface) and between gray matter and cerebrospinal fluid (pial surface).^68^ Cortical thickness was measured in the native space of each individual as the shortest distance between the white and the pial surfaces.^69^ To compare cortical thickness values between groups, cortical surfaces of each subject were registered to the *fsaverage* template and smoothed using a fill-width at half-maximum kernel of 10 mm.

### Statistical analyses in the human sample

Demographic and behavioral data in the human cohort of patients with 22q11DS were analyzed using general linear multivariate analyses of variance with ‘genotype’ (Val or Met) and ‘sex’ (male, female) as main factors and chi-square (*χ*^2^) tests. The softwares STATISTICA (StaSoft, 12) and SPSS (Version 22.0, IBM, Armonk, NY, USA) were used. Based on the available data, in the neuroimaging and CPT analyses only the Geneva cohort was included, while for the WCST only the Rome cohort. MRI data were analyzed with the Query, Design, Estimate, Contrast tool in FreeSurfer, including age, sex and scan type as covariates. This tool provides comparisons of cortical thickness for each vertex of the cortical surface. Therefore, we reported in the results section the higher values of F and the smaller values of *P* for the nonsignificant results and the opposite for the significant ones. Three different analysis were conducted using a general linear model. First, we tested on the entire group of patients (pre- and post-pubertal subjects) the presence of a main effect of genotype and the interaction between genotype–sex and genotype–puberty. We could not conduct a longitudinal analysis because only 18 subjects had a time point in both pre- and post-puberty. For these 18 subjects only the pre-pubertal time point was kept for this first analysis because there we had less subjects. Second, we split the subjects into a pre-pubertal and a post-pubertal group and tested the effect of the *COMT* genotype, using the genotype as factor, as well as the interaction between genotype and sex. The third analysis was conducted in the group of post-pubertal subjects only. In this analysis, males and females were considered separately and, as before, the effect of the *COMT* genotype was investigated using the genotype as factor. As we considered males and females separately, we did not include sex as covariate in this analysis. A Monte Carlo simulation with a *P*-value threshold at *P*<0.05 was used to correct for multiple comparisons.^70^

### Mouse studies

#### Subjects

All procedures were approved by the Italian Ministry of Health (permit no. 230/2009-B) and local Animal Use Committee and were conducted in accordance with the Guide for the Care and Use of Laboratory Animals of the National Institutes of Health and the European Community Council Directives. *COMT* null mutant mice (COMT−/−), their heterozygous (COMT+/−), and wild-type (COMT+/+) littermates with a C57BL6J background were bred by heterozygous (+/−) mating and were identified by PCR analysis of tail samples. The mice were weaned at postnatal day (P) 28 and they were group-housed in a climate-controlled animal facility (22±2 °C) and maintained on a 12 h light/dark cycle, with free access to food and water in individually ventilated cages. The testing was conducted during the light phase. The experimenter was blind to the genotype during testing. In the current work, P2 to P14 was considered as infancy in mice, whereas adolescent mice were tested at 35±1 days, mice from 3 to 6 months were considered adults. The day of birth was defined as P0. Different cohorts of naive mice were used for each single experiment.

#### Structural magnetic resonance imaging in mice

High-resolution morpho-anatomical T_2_-weighted (T_2_W) structural MRI scans were acquired using a 7.0 Tesla scanner at the IIT center in Rovereto, Italy. We adopted sample preparation and MRI acquisition procedure that permits to obtain artifact-free high-resolution images devoid of physiological or motion artifacts as we recently described.^31, 71^ Extensive details are reported in Supplementary Methods.

#### Voxel-based morphometry

Intergroup differences in local gray matter volume were mapped with voxel-based morphometry (VBM)^72^ using ANTs.^73^ In recent work we showed that foci of increased gray matter as detected with VBM correspond to increased cortical thickness as measured postmortem in histopathological assessments.^31^ This correspondence supports the use of VBM as a surrogate for cortical thickness in lissencephalic species like the laboratory mouse (more details in Supplementary Methods). To provide an illustrative description of inter-group effect size between genotypes, mean gray matter volume was quantified in representative symmetric 7x7x7 voxel regions centered over foci exhibiting significant inter-group differences.^74^

#### Antibodies and western blot analyses

The anti-phosphoAkt (Thr308, #2965; Ser473, #9271) and anti-Akt (#2920) were purchased from Cell Signaling Technology (Beverly, MA, USA); while the anti-tyrosine hydroxylase (TH, sc-25269) was purchased from Santa Cruz Biotechnology (Heidelberg, Germany), and the anti-actin antibody (A2066) from Sigma Aldrich (Milan, Italy). The mice were killed by decapitation. The PFC was rapidly dissected on an ice-cold surface and frozen in dry ice before protein extraction. More details are reported in the Supplementary Methods.

#### Stereological developmental analyses

For stereological counts, the number of NeuN positive cells in the inner layers of the pregenual medial PFC (mPFC), consisting of cingulate, prelimbic and infralimbic regions (Supplementary Figure 1 and Supplementary Methods for more details), was evaluated using a stereological fractionator sampling design with the optical fractionator probe of the Stereoinvestigator software as previously described.^75^ The number of NeuN positive cells was counted by an experimenter blind to the experimental group. Sections collection and preparation followed standard protocols as reported in ref. 31.

#### Behavioral procedures

A detailed description is reported in the Supplementary Methods. Developmental milestones were assessed every 2 days from P2 to P14 according to Scattoni’s protocol.^76^ The locomotor activity and temporal order object recognition memory tasks were performed as previously described.^77, 78^

#### Statistical analysis in the mice sample

Results are expressed as mean±standard error of the mean (s.e.m.) throughout. Mice MRI, indices of pups’ growth and development, locomotor activity, object exploration, markers of cellular (Hoechst+), neuronal (NeuN+), and dopaminergic system (TH and Akt) were all examined using general linear multivariate analyses of variance with ‘genotype’ (COMT +/+, +/−, −/−) and ‘sex’ (male, female) as main factors. Temporal order object recognition discrimination index testing whether the time investigating the fewer recent object differed from chance level was assessed using one sample *t*-tests. *Post hoc* analyses for individual group comparisons were carried out with Newman–Keuls *post hoc* test with multiple comparisons corrections, when statistical significance emerged in the main effects or interactions. The accepted value for significance was *P*<0.05. The software STATISTICA (StaSoft, 12) was used.

### Sample size

For animal studies, the target number of samples in each group was determined based on equivalent tests previously published. For both humans and animals experiments, no statistical methods were used to predetermine sample size.

### Replication

Results from western blot experiments were replicated three times. Behavioral experiments and cell counting in mice were replicated four to five times.

### Randomization

For behavioral and histological analysis, age- and sex-matched animals were randomly chosen from our colony. No explicit randomization algorithm was used.

### Exclusion criteria

For behavioral studies, only one adolescent female COMT+/− mouse was excluded as it died before the day of temporal order object recognition experiment for unknown reasons. For human studies, no data points were excluded.

## Results

### In 22q11DS, female COMT Met carriers show reduced cortical thickness after puberty

**Figure 1:**
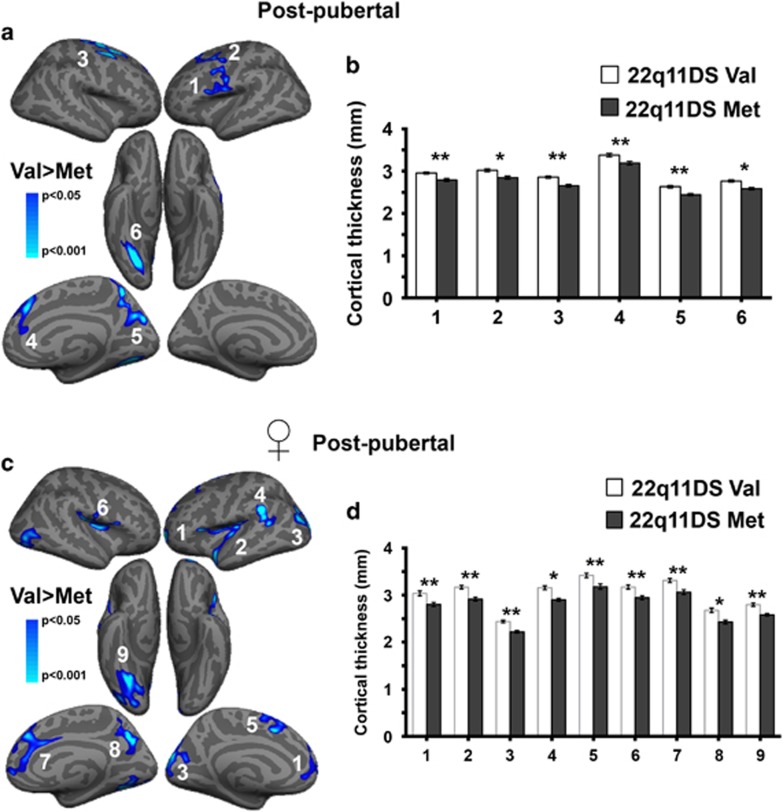
22q11DS *COMT* Met carriers have reduced cortical thickness compared with Val carriers only after puberty and especially in females. (**a**) A significant reduction of cortical thickness in 22q11DS Met carriers was observed compared with Val carriers in post-puberty. There were no clusters of greater thickness in Met compared with Val carriers. (**b**) Mean cortical thickness values for the clusters 1 to 6 are reported in the plots. (1=left precentral/inferior middle frontal cortices, 2=left superior frontal cortex, 3=right superior frontal cortex, 4=right medial superior frontal cortex, 5=precuneus, 6=posterior fusiform gyrus). Post-pubertal: *COMT* Val *N*=34; *COMT* Met *N*=36. *P<0.05 and **P<0.01 versus 22q11DS Val carriers. (**c**) When considering only females post-pubertal patients, a significant reduction of cortical thickness in 22q11DS Met carriers compared with Val carriers was similarly observed. Cortical thickness did not significantly differ in male patients carrying the Met and Val *COMT* allele. (**d**) The plots represent mean cortical thickness values in female patients for each cluster. (1=left superior frontal cortex, 2=left inferior frontal/insula/superior temporal cortices, 3=left occipital cortex, 4=left supramarginal gyrus, 5= left medial superior frontal cortex, 6=right inferior frontal cortex, 7=right medial superior frontal/anterior cingulate cortices, 8=right precuneus, 9=right posterior fusiform gyrus/lateral occipital cortex).*P<0.05 and **P<0.01 versus 22q11DS Val carriers.

When considering the entire group of patients with 22q11DS, no significant effects of the COMT genotype (F_1,89_<23.79, *P*>0.05) or significant interactions between genotype–sex (F_1,89_<20.65, *P*>0.05) and genotype–puberty (F_1,89_<19.33, *P*>0.05) were observed. However, the relatively small sample size of the experimental groups, the different pharmacological status between pre- and post-pubertal subjects (Supplementary Tables 1 and 2), as well as the cross-sectional nature of our assessment limited the power of this analysis. Indeed, separate analyses conducted in the groups of pre- and post-pubertal patients revealed that cortical thickness did not differ between *COMT* Met and Val carriers before puberty (Supplementary Figures 2A and B; F_1,37_<16.04, *P*>0.05). Conversely, cortical thickness values were significantly reduced in Met patients compared to Val after the puberty onset in the left precentral cortex, spanning to the inferior and middle frontal cortices and, in the right hemisphere, in the superior frontal cortex (lateral and medial), precuneus and posterior fusiform gyrus (Figures 1a and b; F_1,68_>9.84, *P*<0.04).

To explore the presence of sex-related differences in the cortical thickness reduction in Met post-pubertal subjects, we repeated the analysis in males and females. No differences were observed between Met and Val males (Supplementary Figures 2C and D; F_1,30_<18.05, *P*>0.05). In contrast, a significant difference was observed in females. In particular, female patients carrying the Met allele showed regions of reduced thickness bilaterally in the superior frontal cortex spanning medially to the right anterior cingulate gyrus, in the inferior frontal cortex comprising the left insula and left superior temporal cortex, in the lateral occipital cortex, in the left supramarginal gyrus and in the right posterior fusiform gyrus and precuneus (Figures 1c and d; F_1,37_>12.76, *P*<0.039). These results start to suggest that cortical thinning associated with reduced COMT activity in post-pubertal subjects might be prominently driven by females.

Demographic characteristics for the patients with 22q11DS revealed no significant differences in sex, age, handedness, psychiatric diagnosis and medications between *COMT* Met and Val subjects (all *P*>0.23; Supplementary Tables 1 and 2). However, in the group of post-pubertal subjects included in the Geneva cohort there was a significant effect of the genotype (*P*=0.046), with less Val subjects meeting the criteria for a psychiatric diagnosis compared with Met subjects.

### Adult female COMT knockout mice show decreased cortical gray matter volume

To directly and more selectively examine the effects of genetic-driven COMT reduction, we next used COMT knockout mice (+/− and −/−) and their wild-type (+/+) littermates. Indeed, our human data might be biased by the unbalance between the numbers of pre- and post-pubertal patients as well as the presence of pharmacological treatments in post-pubertal but not in pre-pubertal patients.

Our previous MRI, VBM analysis of gray matter in mice showed that *COMT* genetic reduction resulted in increased fronto-cortical and postero-parieto-temporal gray matter in males, an effect that was tightly associated with local cortical thickness increases as measured with histopathological assessments.^31^ This supports the use of VBM as a surrogate for cortical thickness in lissencephalic species like the mouse, while in human imaging genetics thickness measurements are preferred.^79^

**Figure 2:**
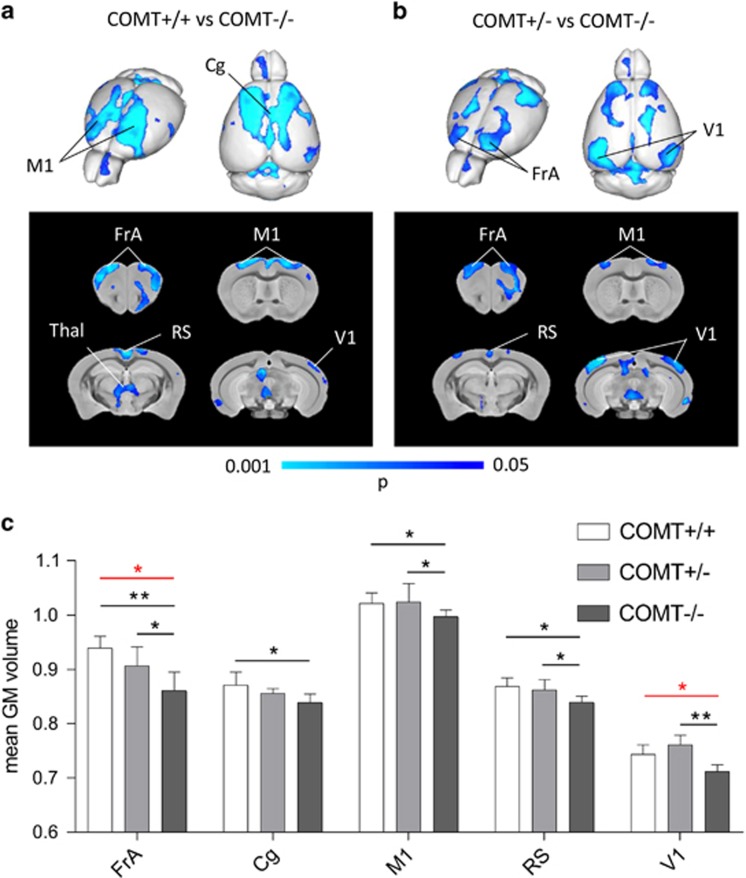
Decreased cortical gray matter volume is evident only in COMT−/− female mice. Structural magnetic resonance imaging (MRI) in COMT knockout female mice. (**a** and **b**) 3D volumetric reconstruction and representative coronal slices of the areas showing statistically significant decreased gray matter (GM) volume. (**a**) Comparisons between female COMT−/− versus +/+. (**b**) Comparisons between GM volume in female COMT+/− versus −/−. (**c**) GM effect size estimation in representative bilateral region of interest. Red asterisks indicate significant F-statistics across genotypes as measured with analysis of variance (ANOVA). Black asterisks indicate significant t-statistics between genotypes. *P<0.05 and **P<0.01. (FrA=frontal association cortex; M1=primary motor cortex; Cg=cingulate cortex; RS=retrosplenial cortex, Thal=thalamus, V1=primary visual cortex). COMT+/+ females *N*=6; COMT+/− females *N*=8; COMT−/− females *N*=5.

In the present work, we re-analyzed the same cohort of mice that underwent VBM analysis in our previous study^31^ to corroborate the role of *COMT* genetic reduction on brain aberrations observed in female patients carrying the low-activity variant. In agreement with the results from 22q11DS human patients (Figure 1), and conversely to male humans and mice,^31^ VBM gray matter mapping revealed bilateral foci of decreased cortical volume in female COMT−/− compared with COMT+/+ (*P*<0.05, threshold free cluster enhancement (TFCE) corrected; Figure 2a), and COMT+/− littermates (*P*<0.05, TFCE corrected; Figure 2b).

No differences were evident between COMT+/+ and +/− mice (*P*>0.06; Figure 2c), indicating that only extreme reduction of COMT can bring female mice to these consistent cortical thinning. Regions particularly affected were frontal associations, primary motor, visual cingulate and retrosplenial cortices, plus additional foci of gray matter decrease in the thalamus (*P*<0.05, TFCE corrected; Figure 2). No foci of significant gray matter volume increase were observed. When these data sets were analyzed on a regional basis using analysis of variance, we found significant F-statistics across genotypes in frontal association and primary visual cortex (Figure 2c).

The new results of cortical thinning in females with 22q11DS and COMT−/− mice are inverse to the cortical thickening we previously observed in post-pubertal healthy men Met carriers as well as in COMT knockout adult males.^31^ Notably, we provide initial biological validation that genetic modifications resulting in excessive reduced COMT activity lead to decreased cortical gray matter selectively in females.

### Tyrosine hydroxylase levels revealed puberty as a turning point in the COMT-by-sex interactive effects

Increased dopaminergic tone might act as a neurotrophic factor influencing cortical development,^80, 81^ and COMT finely modulates dopamine levels in PFC in adulthood.^5, 82^ To examine how reduced COMT might differentially modulate the development of the cortical dopaminergic system in a sex-specific manner, we first analyzed in the PFC the protein levels of the rate limiting step in the dopamine synthesis: TH.^83^

**Figure 3:**
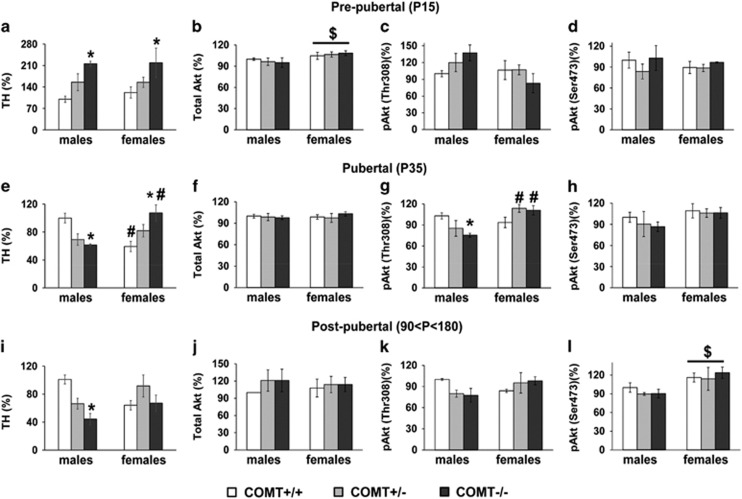
Dopamine-related biochemical markers indicate puberty as the starting point of the appearance of *COMT*-by-sex developmental dichotomy. Densitometric analysis of tyrosine hydroxylase (TH) in the PFC of COMT +/+, +/− and −/− male and female mice at three different stage of development, (**a**) pre-puberty: P15, (**e**) puberty: P35 and (**i**) post-puberty: 5–6 months old. Actin was used as a loading control and did not differ between groups in each developmental stage. Expression levels were normalized to the male wild-type group. Pre-pubertal COMT+/+ males *N*=6, females, *N*=6; COMT+/− males *N*=8, females *N*=7; COMT−/− males *N*=3, females *N*=3. Pubertal COMT+/+ males *N*=8, females, *N*=8; COMT+/− males *N*=13, females *N*=12; COMT−/− males *N*=5, females *N*=9. Post-pubertal COMT+/+ males *N*=5, females, *N*=6; COMT+/− males *N*=5, females *N*=9; COMT−/− males *N*=6, females *N*=5. *P<0.05 versus COMT +/+ within the same sex, ^#^*P*<0.05 versus male within the same genotype. Densitometric analysis of total Akt levels (**b**, **f** and ** j**), phosphoAkt (pAkt, THr308) (**c**, **g** and **k**), and pAkt (Ser473) (**d**, **h** and **l**) in PFC of COMT +/+, +/− and −/− male and female mice at three different stage of development, (**b**–**d**) pre-puberty: P15, (**f**–**h**) puberty: P35, and (**j**–**l**) post-puberty: 5–6 months old. For the measurement of total protein levels, actin was used as loading controls; whereas, for the measurement of phospho-protein levels, total protein levels were used as loading controls. Expression levels were normalized to the male wild-type group. Pre-pubertal COMT+/+ males *N*=6, females, *N*=6; COMT+/− males *N*=6, females *N*=6; COMT−/− males *N*=4, females *N*=3. Pubertal COMT+/+ males *N*=7, females, *N*=5; COMT+/− males *N*=5, females *N*=6; COMT−/− males *N*=6, females *N*=7. Post-pubertal COMT+/+ males *N*=4, females, *N*=5; COMT+/− males *N*=3, females *N*=3; COMT−/− males *N*=4, females *N*=6. *P<0.05 versus COMT +/+ within the same sex, ^#^*P*<0.05 versus male within the same genotype, ^$^*P*<0.05 versus males. PFC, prefrontal cortex.

In the pre-pubertal period (P15), when the sexual hormones levels are lower than post-puberty, we did not see any sex difference in the TH protein level (F_1,26_=0.14, *P*=0.7). During this time window, only a genotype effect was evident (F_2,26_=6.49, *P*<0.005). In particular, COMT−/− mice showed increased TH levels compared with wild-type mice (*P*<0.005; Figure 3a), independently from the sex of the subject.

During puberty (P35), when the sexual maturity is reached,^84^ we observed a different pattern of TH protein levels. Indeed, a strong *COMT*-by-sex interaction was now evident (F_2,70_=7.92, *P*<0.005). Although *COMT* genetic reduction decreased the TH levels in males (*P*<0.05; Figure 3e), the opposite was true in females (*P*<0.05; Figure 3e). Thus, females kept having the same ‘pre-pubertal’ phenotype, whereas males showed a completely opposite phenotype. Furthermore, wild-type females presented reduced TH levels compared with the wild-type males (*P*=0.05; Figure 3e).

In post-pubertal mice (90<*P*<180), an effect of *COMT*-by-sex interaction was still present (F_2,70_=7.92, *P*=<0.001, Figure 3i). In particular, as in puberty, adult male COMT−/− showed lower TH levels compared with +/+ males (*P*<0.05). In contrast, adult females presented an equal amount of TH among the three genotypes (*P*=0.98). Representative western blots for all analyses are reported in Supplementary Figure 3. The results obtained in adult male mice are in line with previous findings reporting that higher COMT activity in males is associated with higher TH protein levels.^7^

Together, these findings indicate that *COMT*-by-sex divergent interactive effects in the cortical dopamine system start to appear during puberty.

### Akt levels and activity confirmed puberty as a turning point in the *COMT*-by-sex interactive effects

To further explore the evidence that the *COMT*-by-sex interaction appear during puberty, we analyzed total and phosphorylated levels of Akt, an intracellular key regulatory protein related to dopamine/D2 effectors.^85^ Moreover, Akt regulates cell proliferation, growth, survival and metabolism, and it has been implicated in sex differences and psychiatric disorders.^5, 86^

Pre-pubertal female mice showed higher level of total Akt than males (F_1,27_=5.73, *P*<0.05; Figure 3b), independently from the *COMT* genotype (F_1,27_=0.03, *P*=0.97). In contrast, no effect of either sex or genotype was evident in the rate of phosphorylation at both the Thr308 (sex: F_1,25_=3.07, *P*=0.09; genotype: F_2,25_=0.33, *P*=0.71; Figure 3c) and Ser473 sites (sex F_1,24_=0.09, *P*=0.7, genotype: F_2,24_=0.49, *P*=0.6; Figure 3d).

In contrast, during puberty, the total Akt protein levels were unaffected by the sex (F_1,35_=0.08, *P*=0.7) and *COMT* genotype (F_2,35_=0.17, *P*=0.8) of the subjects (Figure 3f). However, a strong sex-by-*COMT* interaction was evident for the phosphorylation at the Thr308 site (F_2,29_=6.56, *P*<0.005; Figure 3g), but not at the Ser473 site (F_2,25_=0.16, *P*=0.8; 3H). In particular, although no difference was detected between males and females COMT+/+ mice (*P*=0.3; Figure 3g), *COMT* genetic reduction decreased pAkt levels exclusively in males (*P*<0.05; Figure 3g). Moreover, COMT+/− and −/− females had much higher levels than males within the same genotype (*P*<0.05; Figure 3g).

A similar sex-by-*COMT* interaction effect in the pAkt levels at the Thr308 site was evident also in post-pubertal mice (F_2,18_=3.8, *P*<0.05; Figure 3k). Representative western blots for all analyses are reported in Supplementary Figure 3.

Higher dopamine corresponds to lower level of Thr308pAkt.^85^ Thus, these data correlate well with the TH levels found in COMT developing mice, further strengthening the conclusion that *COMT*-by-sex interactive effects in the cortical dopamine system start to appear during adolescence.

### Sex-dichotomous effects of *COMT* genetic mutations on PFC neuronal counting in mice arise at puberty

We previously found that COMT knockout adult males exhibited an increase in neuronal density in the mPFC layers V/VI, but not in layers II/III.^31^ Conversely, COMT knockout adult females exhibited a decreased neuronal density in the mPFC layers V/VI, but not in layers II/III.^31^ Here we asked whether *COMT* genetics might affect the absolute neuronal and cellular counting in the mPFC (Supplementary Figure 1) throughout the lifespan and if and when *COMT* sex-dependent effects start to appear. We adopted a stereological unbiased approach to estimate the total number of neurons in the mPFC in pre-pubertal, pubertal and post-pubertal mice.

**Figure 4:**
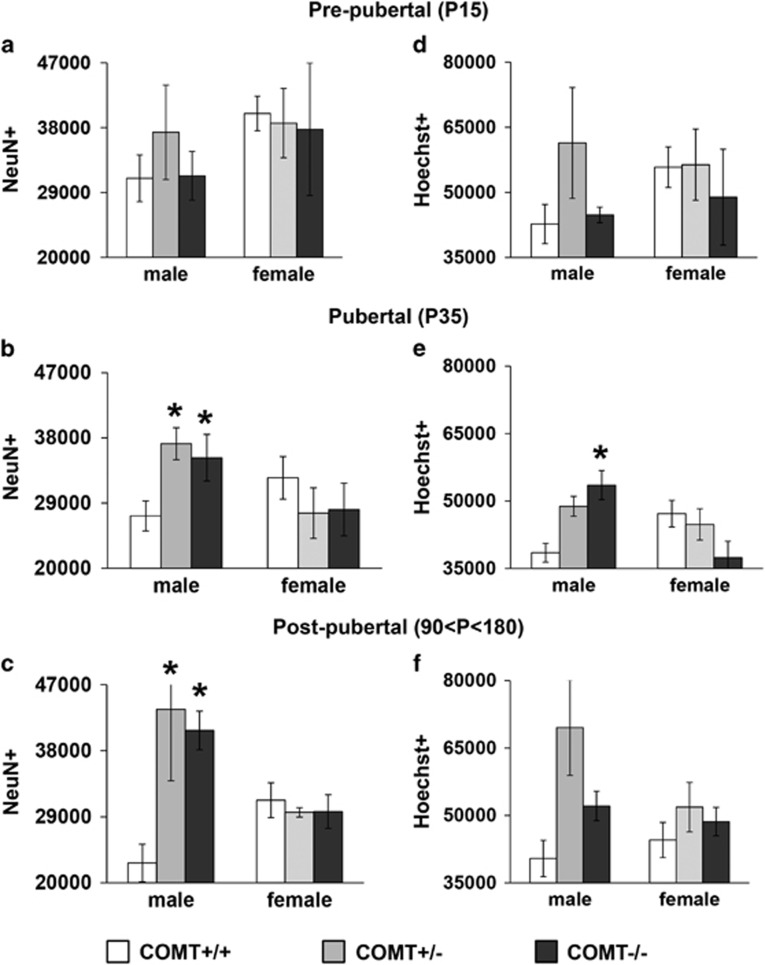
Sexually divergent effects of *COMT* genetic disruption on neuronal and total cells counting in deep layers of the medial prefrontal cortex (mPFC) arise during adolescence. *COMT* genetic disruption does not affect neuronal counting (NeuN+) in infant mice (**a**), while a sex-by-genotype interaction is shown in adolescent (**b**) and adult (**c**) mice. *COMT* genetic deletion does not affect cellular counting (Hoechst+) in (**d**) infant mice and (**f**) adult mice, whereas it increases the total number of cells in (**e**) adolescent males. Pre-pubertal COMT+/+ males *N*=4, females, *N*=4; COMT+/− males *N*=3, females *N*=5; COMT−/− males *N*=4, females *N*=3. Pubertal COMT+/+ males *N*=5, females, *N*=4; COMT+/− males *N*=5, females *N*=4; COMT−/− males *N*=5, females *N*=4. Post-pubertal COMT+/+ males *N*=5, females, *N*=6; COMT+/− males *N*=3, females *N*=3; COMT−/− males *N*=5, females *N*=4. *P<0.05 versus COMT+/+ of the same sex.

In pre-pubertal mice (P15), analysis on neuronal estimate (NeuN positive cells) did not show any significant difference for sex (F_1,15_=1.56, *P*=0.23), *COMT* genotype (F_2,15_=0.22, *P*=0.8) or their interaction (F_2,15_=0.31, *P*=0.74; Figure 4a). Similarly, the total number of cells (Hoechst positive) was not affected by sex (F_1,15_=0.48, *P*=0.5), *COMT* genotype (F_2,15_=1.2, *P*=0.3), or their interactions (F_2,15_=0.87, *P*=0.4; Figure 4d).

In contrast, a *COMT*-by-sex interaction effect on the number of neurons emerged at puberty (F_2,20_=3.8, *P*=0.03; Figure 4b), while no effect of sex (F_1,20_=2.56, *P*=0.13) or genotype (F_2,20_=0.42, *P*=0.66) was evident. In particular, COMT−/− and +/− male mice (*P*<0.04; Figure 4b), but not females (*P*=0.5; Figure 4b), had more neurons than COMT+/+. Similarly, for the total number of cells, we detected a significant *COMT*-by-sex interaction (F_2,20_=3.6, *P*=0.004). In particular, COMT−/− males (*P*<0.05; Figure 4e), but not females (*P*=0.4; Figure 4e), had a higher number of cells compared with +/+ mice.

Finally, in post-pubertal mice, as expected, we found a significant *COMT*-by-sex interaction effect (F_2,19_=6.7, *P*=0.006). In particular, *post hoc* analysis revealed that COMT+/− and −/− males had a higher number of neurons compared with COMT+/+ in the mPFC (*P*<0.05; Figure 4c). Furthermore, for the total amount of cells, we observed a significant effect of genotype (F_2,19_=5.8, *P*=0.01), but not of sex (F_1,19_=2.0, *P*=0.2) or genotype-by-sex interaction (F_2,19_=2.1, *P*=0.1; Figure 4f).

Taken together, these results indicate that *COMT*- and *COMT*-by-sex interaction effects start to appear during adolescence, are absent before puberty and persist in adulthood.

### Genetic-driven COMT reduction in mice did not alter developmental milestones and locomotor functions from birth to adulthood

Based on the consistent molecular and anatomical data showing that *COMT*-by-sex interacting effects start to appear during adolescence, we next checked whether similar pattern of effects might be evident in behavioral outputs.

Genetic reduction or absence of COMT in mice did not affect developmental trajectories of somatic growth indexes, general health, physical maturation and locomotor functions in either sexes from birth up to adulthood (Supplementary Results, Supplementary Figure 4 and Supplementary Table 3).

Taken together, these results indicate that *COMT* genetic reduction does not alter pups’ development nor interact with the sex of the subjects for gross behavioral maturation from early postnatal periods, across puberty and up to adulthood.

### Sex-dichotomous *COMT* genetic effects in cognitive functions are apparent from adolescence in mice

*COMT* genetic variants impact cortical development in a sex-dichotomous way starting from adolescence (Figures 1). *COMT*-by-sex-dichotomous effects in PFC-dependent cognitive abilities are evident in adults.^7, 31^ Thus, we addressed when these *COMT*-by-sex interacting effects might appear during development. The T-maze task that we previously used in adult mice^7, 31^ is not suited to test developing mice in a specific age point as it requires long training, dietary restraint and stressful manipulation. We then took advantage of the temporal order object recognition task, a cognitive test for mice that requires a functional PFC, perhirinal cortex and hippocampus and that depends on cortical dopamine signaling.^77, 87^

Pre-pubertal mice (P15) did not show any discrimination ability in the temporal order object recognition task (data not shown),^88^ consistent with a still immature cortical development.^89^

**Figure 5:**
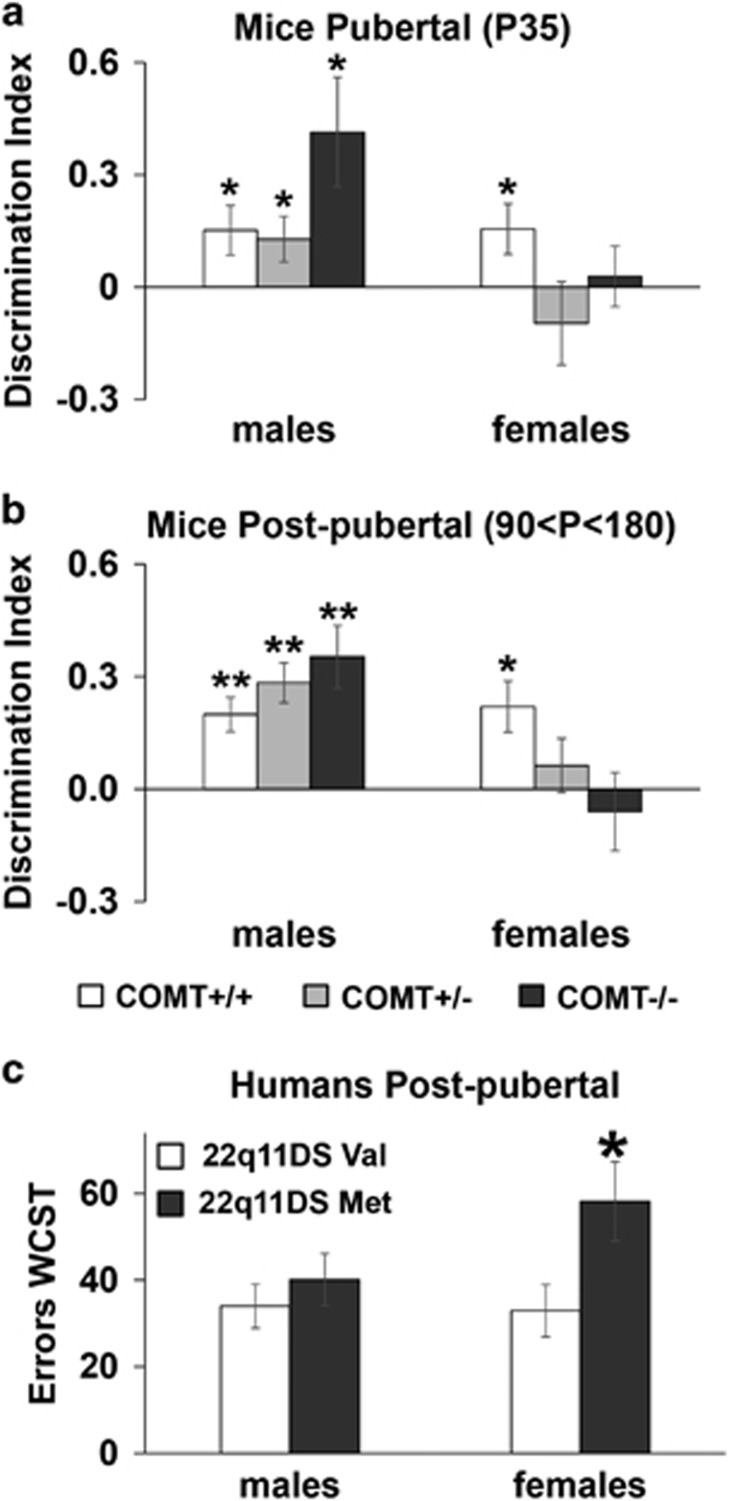
*COMT* sexually divergent effects on cognitive functions are evident from adolescence in both mice and human patients with 22q11DS. Discrimination index displayed by (**a**) adolescent and (**b**) adult COMT+/+, +/− and −/− males and females littermates mice during the 5 min temporal order object recognition test. Pubertal COMT+/+ males *N*=12, females, *N*=14; COMT+/− males *N*=17, females *N*=12; COMT−/− males *N*=6, females *N*=6. Post-pubertal COMT+/+ males *N*=16, females, *N*=11; COMT+/− males *N*=11, females *N*=18; COMT−/− males *N*=9, females *N*=8. *P<0.05 and **P<0.005 versus chance levels. (**c**) Number of errors made during the Wisconsin Card Sorting test (WCST) by subjects affected by 22q11DS divided by their sex (males or females), and COMT genotype (Val or Met). COMT Val males *N*=22, females, *N*=12; COMT Met males *N*=20, females *N*=12. *P<0.05 versus all other groups.

Cognitive performance of pubertal and post-pubertal mice revealed a strong sex-by-genotype interacting effect (F_2,128_=6.0, *P*=0.003). Indeed, COMT+/− and −/− females had a worse cognitive performance compared with females COMT+/+, and males +/+ and −/− (*P*<0.05; Figures 5a and b). More specifically, in pubertal mice, all males groups (COMT+/+, +/− and −/−) showed a positive discrimination index as they explored the object presented least recently more than chance levels (*P*<0.05; Figure 5a). This indicated intact recency memory abilities in COMT knockout males. If anything, COMT−/− males showed a tendency towards an improved performance compared with +/+ males (*P*=0.07). In contrast, in females, only the wild-type group showed a positive discrimination index (*P*<0.05), whereas both COMT+/− and −/− were not able to distinguish between the two objects (*P*>0.42). Thus, *COMT* genetic reduction impaired this cognitive function only in pubertal females.

Similarly, in adult mice, all males groups (COMT+/+, +/− and −/−) showed a positive discrimination index (*P*<0.003; Figure 5b), indicating that mice were able to discern between the object presented least recently than the object presented most recently. Again, COMT−/− males tended to outperform +/+ male littermates (*P*=0.08). Conversely, in females, only the wild-type group showed a positive discrimination index (*P*<0.01), while both COMT+/− and −/− were not able to distinguish between the two objects (*P*>0.40; Figure 5b).

In both pubertal and adult mice, the total amount of time spent exploring objects in the different experimental phases showed no effects of genotype, sample phase or sample phase-by-genotype interaction (Supplementary Figure 5). Thus, *COMT* genetic reduction did not alter motivation, curiosity, motor, olfactory, tactile or visual functions that might affect object recognition. These findings confirm at the behavioral cognitive level that *COMT*-by-sex interactive effects are already apparent during adolescence.

### Sex-dichotomous *COMT* genetic effects in executive functions are apparent from adolescence in patients with 22q11DS

To investigate whether the COMT sex-dichotomous effects predicted by our mouse studies in cognitive functions would be observable also in patients with 22q11DS, we analyzed their performance in the Wisconsin Card Sorting task. The Wisconsin Card Sorting task is a neuropsychological test commonly used to evaluate PFC-dependent executive control.^90^ A significant *COMT* ValMet-by-sex interaction effect was evident (F_1,61_=3.8, *P*=0.05). *Post hoc* analyses revealed that Met females made more errors compared with all the other groups (*P*<0.05; Figure 5c). This is similar to the cognitive findings from *COMT* genetically modified mice.

In agreement to previous studies in healthy subjects, patients with schizophrenia and genetically modified mice,^7, 9, 31, 32, 91, 92, 93, 94^ COMT-dependent effects were selective to executive functions. Indeed, no COMT or COMT-by-sex interaction effects were evident in general intelligence (IQ), cognitive inhibition (Stroop), attention (CPT) and short-term memory (digit span test; Supplementary Figure 6).

## Discussion

In the present study, we demonstrated that *COMT* genetic variations produce cortical alterations in a sex-specific manner starting from puberty. This was similarly evident in patients with 22q11DS and in COMT knockout genetically modified mice. Notably, *COMT*-dependent anatomical changes in the cortex, and correlated cognitive functions, were paralleled by altered cortical developmental trajectories of molecular markers of the dopamine system. Again, these *COMT*-by-sex molecular rearrangements emerged during adolescence.

Studies in humans and genetically modified mice have shown *COMT*-by-sex interacting effects on cortical morphology^31^ and cognitive functions.^9, 29, 31^ However, the developmental period during which this interaction becomes evident remained unclear. Only few studies investigated the effect of *COMT* genetics on working memory and brain morphology during development,^13, 15, 17^ showing increased cognitive performance with age^15^ and faster rates of cortical thinning^17^ in MetMet subjects, as well as potential *COMT*-by-puberty interaction in Met homozygous males.^13^ In our translational study combining results from patients with 22q11DS and *COMT* genetically modified mice, we demonstrated that puberty is the developmental period when *COMT*-by-sex interactions start to appear, influencing cortical morphology as well as related cognitive functions. Indeed, we found several clusters of reduced gray matter volume in adult COMT−/− female mice, in cingulate, retrosplenial, dorso-frontal cortical areas, recapitulating anatomical features of some of the clusters of reduced cortical thickness in female 22q11DS Met carriers. Similarly, in both adolescent and adult mice, but not in pre-pubertal mice, we also detected sex-dimorphic effects of COMT in the PFC in terms of number of neurons and molecular markers involved in the postnatal maturation and function of the dopaminergic system. The same pattern of effects was evident in cortical-dependent cognitive tasks. Overall, these data show that excessive reduction of COMT, as can be found in Met 22q11DS patients and COMT−/− mice, was deleterious after puberty onset in females but not in males.

Previous studies addressing *COMT*-related differences in 22q11DS reported heterogeneous and fragmented findings. The Met allele has been associated with better cognitive performance and no cortical alterations in children.^50, 95^ In contrast, in adolescent and adult patients, the Met allele has been associated with poorer cognitive abilities,^48, 51, 52^ reduced cortical volume^35, 96^ and increased vulnerability for psychiatric symptoms.^35, 96, 97^ Also, *COMT*-by-sex interactions have previously been showed in 22q11DS.^27, 28, 30^ In particular, Kates *et al.*^30^ have shown that Met girls and Val boys with 22q11DS have increased volume in the dorsal PFC, but reduced volume in the orbital PFC. Coman *et al.*^28^ showed that female patients carrying the Val allele and male patients carrying the Met allele had increased activation in frontal brain regions during the processing of pleasant stimuli, whereas Met females and Val males had an increased activation in limbic regions during the processing of unpleasant stimuli. Furthermore, Boot *et al.*^27^ reported evidence suggesting an effect of sex-by-*COMT* interactions on dopaminergic markers. In the current study, the use of genetically modified mice was important in suggesting which effects were selectively dependent on the extreme *COMT* genetic disruption present in 22q11DS Met female subjects. In particular, avoiding the interacting effects of other genetic variations in and outside the 22q11 deleted region, overcoming the relatively small human samples size, the different pharmacological status between pre- and post-pubertal patients, and the cross-sectional nature of the measurements instead of longitudinal. Furthermore, taking into account the different developmental stages of mice and humans, our findings support the conclusion that the worsening of brain and cognitive alterations observed in post-pubertal patients with 22q11DS are caused, at least in part, by COMT genetic reduction in female subjects.

The molecular mechanisms underlying these developmental sex-dependent *COMT* effects might be complex. We report initial evidence from molecular indicators of the dopaminergic system suggesting that reduced COMT modulates in a very selective age- and sex-dependent way the development and maintenance of the cortical dopamine system. In infancy, the genetic reduction of COMT enhanced TH protein levels independently from the sex of the subjects. This suggests that low levels of COMT, reasonably via cortical dopamine increase, might support the postnatal development of the dopamine system by inducing upregulation of TH enzyme, required for dopamine biosynthesis, and Akt signaling, involved in neuronal survival. Also in the PFC of humans, it has been shown a peak of very high levels of TH during infancy and toddlerhood,^98^ highlighting the large synthetic demand for dopamine early in life when cortical pyramidal neurons are still maturing^26^ and inhibitory interneurons are still migrating and differentiating.^99^ Conversely, from adolescence to adulthood, we found a completely opposite COMT-dependent pattern of TH expression in males while females kept on showing the same phenotype as during infancy. This pattern of changes in TH and Akt in male mice from infancy to adolescence was similar to the switch we found from pre- to post-puberty in cortical thickness in male patients with 22q11DS. Future studies will be required to address the functional relevance of these abrupt changes and how they are related to sexual maturity. However, the negative feedback that might aim to balance the increase of synaptic dopamine and cortical thickness in COMT knockout males is consistent with previous findings reporting compensatory mechanisms in PFC TH levels following COMT-dependent dopamine modulation.^7, 82^ Instead, this compensatory effect in adolescent COMT knockout females might be prevented by the synaptic overdrive of dopamine due to pubertal increase of estrogen levels.^100^ The pAkt data further support this view. In particular, *COMT*-by-sex interactive effects in adolescence were selectively evident in the level of phosphorylation of Akt at the thr309 site, which is directly correlated with the activity of D2 postsynaptic receptors.^85^ This evidence suggests an implication of dopamine/D2 pathways. Of note, Akt pathways are altered in patients with schizophrenia and haloperidol, a D2 antagonist with antipsychotic properties increase the phosphorilation of Akt in wild-type mice.^86^ Overall, these molecular findings strength our morphological and behavioral evidence that *COMT* genetics effects appear during puberty. Puberty is a critical time in relationship to sexual hormones.^101, 102^ Thus, the mechanism underlying *COMT*-by-sex interaction might depend on estrogen and testosterone hormonal levels. Although we cannot ignore the possible implication of testosterone in the phenotypes of males, here we speculated on the possible involvement of estrogens in females phenotypes. Indeed, we recently showed that sex-dichotomous effects of functional *COMT* genetic variations on cognitive functions disappear after menopause in women.^92^ Moreover, endogenous physiological fluctuation of estradiol in healthy women differentially modulated working memory performance based on the *COMT* ValMet genotype.^32^ Estradiol enhances dopamine activity, synthesis, release and turnover.^100, 103^ Indeed, baseline extracellular PFC dopamine levels, mesocortical dopaminergic cells and the release of dopamine after stimulation are all higher in women than in men.^104, 105, 106^ Furthermore, a recent paper^107^ demonstrated a role for progesterone on PFC dopamine maturation and behavioral cognitive performance. Thus, our findings suggest that in the context of extremely low COMT levels, the potential dopaminergic overdrive in post-pubertal/pre-menopausal females has detrimental effects in brain morphology and cognition.

In conclusion, we demonstrated that genetic *COMT* effects in brain structure, behavior and cortical expression of dopamine-related proteins are not static throughout development and are influenced by sexual maturation. In particular, we clarified that *COMT* genetic variations modulate the maturation of brain and behavior from infancy, to adolescence and adulthood in a distinct way in males and females. This information will be crucial to elucidate previous inconsistent findings on *COMT*-dependent effects. Moreover, it provides the background to implement more effective and personalized therapeutic treatments targeting the dopaminergic system based on the *COMT* genotype, the sex and the developmental stage of each person.
