# Supplementary material for: Adolescence is the starting point of sex-dichotomous COMT genetic effects
Source: Transl Psychiatry. 2017 May 30;7(5):e1141–. doi: 10.1038/tp.2017.109 (PMC5584523; doi:10.1038/tp.2017.109)
Supplement: Supplementary Information [file tp2017109x1.docx]

**Supplementary Results**

**Psychiatric diagnosis of patients with 22q11DS**

After semi-structured interview, twenty-six (67%) pre-pubertal subjects met the criteria for one or more psychiatric diagnosis: 2 subjects were diagnosed with attention deficit hyperactivity disorders (ADHD), 14 with anxiety disorders, 1 with a psychotic disorder and 9 with a combination of psychiatric disorders. In the post-puberty group, 45 subjects (63%) met criteria for one of more psychiatric diagnosis: 2 subjects received a diagnosis of ADHD, 12 of anxiety disorders, 5 of mood disorders, 7 of psychotic disorders and 19 met the criteria for more than one psychiatric disorder. One pre-pubertal and 26 post-pubertal subjects were under psychotropic medication at the time of assessment. Nine participants (6 females) were on methylphenidate, 4 (4 females) on antidepressants, 3 (1 female) on antipsychotics, 1 subject (1 female) on anxiolytics, 1 (1 female) on antiepileptic medication, and 8 (1 female) were taking more than one medication (Supplementary Table 1).

**Genetic-driven COMT reduction in mice did not alter developmental milestones from birth to puberty**

Based on the consistent molecular and anatomical data showing that COMT and COMT-by-sex interacting effects start to appear during adolescence (Fig. 1-4), we next checked if similar pattern of effects might be evident in behavioral outputs.

A first analysis of markers of somatic growth revealed that body weight, body length and tail length measured from postnatal days 2 to 14 (i.e. from birth to childhood), did not differ depending on the COMT genotype, the sex of the subject or COMT-by-sex interaction (Supplementary Fig. 3A-C; statistical analyses are reported in Supplementary Table 2).

Similarly, females and males COMT+/+, +/- and -/- pups did not differ in forelimb placing, grasping and righting reflexes (Supplementary Fig. 3E-F and Supplementary Table 2). Only a slight advantage of COMT full deletion was evident for the negative geotaxis reflex. In particular, a COMT-by-sex-by-postnatal day interaction effect (F_12,216_=1.9; p=0.03) revealed that at P4 in males and at P6 in females, COMT-/- required less time to turn to either side when placed head down on a wire mesh screen (p=0.001; Supplementary Fig. 3G and Supplementary table 2).

Furthermore, the measuring of the muscle strength by the bar holding test between P8 and P14 (because no pups were able to hold up a bar before P8) revealed no significant effects of COMT or sex or their interaction. Similarly, in the cliff avoidance test, eye opening, ears detachment from the skull, acoustic reflex, central incisors eruption through the gum, fur appearance and development we did not find any significant effect of COMT, nor a significant COMT-by-sex-by-repeated measure interaction (Supplementary Fig. 3I-M and Supplementary Table 2).

Taken together, these results indicate that COMT genetic reduction does not alter pups development nor interact with the sex of the subjects from early post-natal periods up to preadolescent stages.

**COMT genetic deletion did not affect basal locomotor activity in adolescent mice**

In the day preceding the TOR task, adolescent and adult mice were exposed to the empty apparatus for one hour and basal locomotor activity was scored. Genetic reduction of COMT does not affect basal locomotor activity and behavior in the novel environment in adolescence similarly to what has been reported in adults ([1](#_ENREF_1)). It has been already shown that COMT reduction does not affect basal locomotor activity in a novel environment in adult mice ([1](#_ENREF_1)). Similarly, no significant interaction occurred for the factors sex and genotype or their interaction over time in adolescent mice, suggesting that COMT reduction and sex do not affect habituation in the open field in adolescence. As expected we found a habituation response to the novel environment as indicated by decreased activity over time, with a statistically significant effect of the factor time (F_11,605_=41.6, p=<0.0001, figures not shown).

We also evaluated time spent immobile and time in the periphery that can be altered as a consequence of anxiety-like or fear-related behavior in a novel environment. Statistical analysis showed a significant effect of time (F_11,605_=3.2, p<0.0001) for time in the periphery and time immobile (F_11,605_=32.6, p< 0.001), while no difference was detected for genotype or sex, nor for their interaction (not shown).

Together, these results show that COMT reduction does not affect basal locomotor activity in a novel environment in male and female adolescent mice, consistent to what has been found in adult mice ([1-3](#_ENREF_1)).

**Supplementary Methods**

*Structural magnetic resonance imaging in mice.* High-resolution morpho-anatomical *T*_2_-weighted (*T*_2_*W*) was performed in paraformaldehyde (4% PFA) fixed mouse brains. Standard sample preparation and MRI acquisition have been recently described (Dodero et al. 2013). This procedure permits to obtain artifact-free high-resolution images devoid of physiological or motion artifacts as we previously described ([4](#_ENREF_4)). Briefly, adult COMT null mutant mice (COMT-/-) and +/+ littermates were anesthetized with an intraperitoneal Avertin injection (375 mg/kg) and perfused intracardially with phosphate buffered-saline (PBS) followed by 4% PFA. Both perfusion solutions were added with a Gadolinium chelate (Prohance, Bracco, Milan, Italy) at a concentration of 10 and 5 mM, respectively, to reduce longitudinal relaxation times. Brains were imaged inside intact skulls to avoid post-extraction deformations. MR images were acquired within 6 days from perfusion at 7.0 Tesla using a 72-mm birdcage transmit coil, and a custom-built saddle-shaped solenoid coil for signal reception with the following imaging parameters: 3D rapid acquisition with relaxation enhancement (RARE) spin-echo sequence, Repetition Time (TR) = 550 ms, echo time = 33 ms, RARE factor = 8, echo spacing 11 ms, and voxel size of 90 µm (isotropic).

*Voxel based morphometry analyses in mice.* A study-based template was created aligning the high-resolution T2W images of the control population to a common reference space via a 12 degrees-of-freedom affine alignment, followed by 5 consecutive symmetric diffeomorphic registrations. Individual T2W images of the two groups of subjects were then registered to the study-based template using affine and diffeomorphic registration. Gray matter of spatially normalized subjects was then segmented using a 6-class segmentation of the study-based template as a prior to initialize the process. The Jacobian determinants of the deformation were then used to modulate the GM probability maps calculated during the segmentation step. The modulation compensates for the deformation introduced after the spatial normalization so that there is no variation of the total amount of gray matter, focusing the analysis on the local volumetric variation of the GM instead of the tissue density. The resulting modulated GM probability maps were then smoothed using a Gaussian kernel with a sigma of 3 voxels for voxel-wise statistical parametric comparison. Voxel-wise cross-subject statistic was performed using a nonparametric permutation test with 5000 permutations as implemented in FSL. Data were corrected for multiple comparisons using a cluster-based threshold of 0.05.

*Antibodies and Western Blot Analyses.* The anti-phospho Akt (Thr-308, #2965; Ser473, #9271) and anti-Akt (#2920) were purchased from Cell Signaling Technology (Beverly, MA); while the anti-tyrosine hydroxylase (TH, sc-25269) were purchased from Santa Cruz Biotechnology (Heidelberg, Germany), and the anti-actin antibody (A2066) from Sigma Aldrich (Milan, Italy). Mice were euthanized by decapitation. PFC was rapidly dissected on an ice-cold surface and frozen in dry ice before protein extraction. Tissue samples were homogenized in boiling 1% SDS solution supplemented with inhibitor of protease and phosphatase (Sigma Aldrich, Milan, Italy) and boiled for 5 min. Protein concentrations were measured using a DC-protein assay (Bio- Rad, Hercules, CA). Protein extracts were separated on precast 10% SDS/PAGE (Biorad, Milan, Italy) and transferred to nitrocellulose membranes. Blots were incubated with primary antibodies overnight at 4°C. Immune complexes were detected using appropriate peroxidase-conjugated secondary antibodies (Thermo Fisher Scientific, IL, USA) and a chemiluminescent reagent (ECL prime; GE Healthcare Europe GmbH, Milan, Italy). Densitometric analysis was performed by ImageQuantTL software (GE Healthcare Europe GmbH, Milan, Italy). For quantitative analysis, total proteins were used as loading controls for phosphoprotein signals, while actin was used for the total proteins. Results were normalized to respective control conditions.

*Stereological developmental analyses.* For stereological counts, the number of NeuN positive cells in the inner layers of the pregenual mPFC, consisting of cingulate (Cg1), prelimbic (PrL), and infralimbic (IL) regions (Supplementary Fig. 1), was evaluated using a stereological fractionator sampling design with the optical fractionator probe of the Stereoinvestigator software as previously described ([5](#_ENREF_5)). The number of NeuN positive cells was counted by an experimenter blind to the experimental group. 40 µm serial coronal sections were collected; every third section was immunostained for NeuN using standard protocol ([4](#_ENREF_4)). Deep layers were examined as our previous study suggests that deep layers V and VI undergo the main COMT*sex effect on cortical thickness and neuronal counting in adult mice ([4](#_ENREF_4)). Each section of the prefrontal cortex was viewed at low power (4 X objective) and the area of interest, corresponding to the deep layers of the medial prefrontal cortex, was outlined using the white matter as anatomic landmark as described previously ([5](#_ENREF_5)), the same anatomical landmark were used for P15, P35 and adult mice. Starting at a random microscope visual field, the number of NeuN positive cells was counted at high power (40 X objective). The coefficient of error (CE) was below 0.1 in all animals studied, indicating that our sampling was consistent. An unbiased counting frame of known area (70 × 70 μm) was superimposed on the image stacks, and counts were made at regular predetermined intervals (x = 250 μm, y = 250 μm) from a random start point, by an experimenter blind to the experimental groups.

*Neurodevelopmental profile of infant mice lacking COMT gene.* Developmental milestones were assessed every two days from P2 to P14, pups were weighed and their body and tail lengths were measured. Fur development, day of eyelid opening, pinnae detachment and incisor eruption were also recorded.

Pups were tested according to Scattoni’s protocol ([6](#_ENREF_6)). The tests were conducted during the light phase of the circadian cycle, between 12:00 and 17:00 h. Each subject was tested at approximately the same time of day. Reflexes and responses were scored in the following order:

1. Forelimb placing reflex: pup raises and places its forepaw on the surface of the edge of an object when stroked on the dorsum of the paw.
2. Forelimb grasping reflex: pup grasps the shaft of a toothpick when the forepaw is stroked.
3. Righting reflex: pup turns over with all four feet on the ground when placed on its back.
4. Negative geotaxis: pup turns approximately 180° to either side when placed head down on a wire mesh screen (4×4 mm) held at a 45° angle.
5. Auditory startle: pup reacts to acoustic stimuli (snapping of the fingers) by a startle response.
6. Bar holding: pup grasps a small wire bar by its forelimbs. Time spent holding the bar was scored, the cut off was set at 10seconds.
7. Cliff avoidance: pup avoids the cliff when placed on the edge of a table.

Latencies were measured in seconds, using a stopwatch for righting reflex, negative geotaxis and bar holding. Other somatic and behavioral variables were rating semi-quantitatively: 0 = no response/reaction, 1 = slight response/reaction, 2 = incomplete response/reaction, and 3 = a complete adult-like response. Unless otherwise noted, absence of a milestone was scored as zero if the mouse did not exhibit the behavior within 60 s.

Pups were tattooed on the paw with animal tattoo ink (Ketchum permanent Tattoo Inks green paste, Ketchum Manufacturing Inc., Brockville ON Canada) by loading the ink into a 30G hypodermic needle and inserting the ink subcutaneously through the needle tip into the center of the paw. The procedure was performed at two days of age, immediately after behavioral testing. The procedure causes only minor brief pain and distress and does not require the use of anesthesia.

*Locomotor activity and temporal order object recognition memory (TOR) task.* The apparatus was a plastic open field arena (40cm x 40cm x 20cm for adolescent mice and 40cm x 40cm x 40cm for adult mice), with opaque walls of 50 cm. An overhead camera and Anymaze software were used to monitor and record animals’ behavior for subsequent analysis. Four arena were available and used at the same time, thus allowing to test four animals at the same time. The stimuli presented were copies of plastic objects: a parallelepiped 2cm x 2cm x 8 cm and a conic flask 5cm (diameter) x 8cm, the objects were filled with black or white sand and were too heavy for the animal to displace. The t*emporal order object recognition memory (TOR) task was performed as previously described (*[*7*](#_ENREF_7)*,* [*8*](#_ENREF_8)*). M*ice were first habituated to the open field arena on day 1 without stimuli for 1 hour. Basal locomotor activity was examined in this phase, in particular, we scored the total distance travelled, time spent immobile, time spent in the periphery of the apparatus. Each parameter was examined in segments of 5 minutes to reveal habituation over time. Temporal object preference test occurred the day after. The procedure comprised two sample phases and a test trial. In each sample phase, the subjects were allowed to explore two identical objects for 5 minutes. Different objects were used for sample phase 1 and 2, with a delay of 1 hour. The test trial was given 3 hours after the end of the sample phase 2. In the test trial, a third copy of the objects from sample phase 1 and 2 was used. The objects were always placed in the center of the apparatus at a distance of about 10 cm from the walls and about 15 cm from the other object, the order in which the objects were presented was randomized and counterbalanced between the animals, colors were alternated. For example, if two black parallelepipeds were used in sample 1 then two white flasks were used in sample 2 and, in the test trial a black parallelepiped and a white flask were used. We expect the subjects will spend more time exploring the less recent object, which is the object presented in sample 1, compared with the object from sample 2 when temporal order memory is intact ([7](#_ENREF_7)). Object exploration was measured during the three phases of the TOR task. Exploration was defined as the animal directing its nose toward the objects at a distance less than 2 cm. Sitting or climbing on the object was not considered as exploration. Discrimination between the objects consisted in more time spent with the less recent object compared to the more recent one.

**References**

1. Gogos JA, Morgan M, Luine V, Santha M, Ogawa S, Pfaff D, et al. Catechol-O-methyltransferase-deficient mice exhibit sexually dimorphic changes in catecholamine levels and behavior. Proceedings of the National Academy of Sciences of the United States of America. 1998;95(17):9991-6.

2. Papaleo F, Crawley JN, Song J, Lipska BK, Pickel J, Weinberger DR, et al. Genetic Dissection of the Role of Catechol-O-Methyltransferase in Cognition and Stress Reactivity in Mice. The Journal of neuroscience : the official journal of the Society for Neuroscience. 2008;28(35):8709-23.

3. Papaleo F, Erickson L, Liu G, Chen J, Weinberger DR. Effects of sex and COMT genotype on environmentally modulated cognitive control in mice. Proceedings of the National Academy of Sciences of the United States of America. 2012;109(49):20160-5.

4. Sannino S, Gozzi A, Cerasa A, Piras F, Scheggia D, Manago F, et al. COMT Genetic Reduction Produces Sexually Divergent Effects on Cortical Anatomy and Working Memory in Mice and Humans. Cerebral cortex. 2015;25(9):2529-41.

5. Manitt C, Mimee A, Eng C, Pokinko M, Stroh T, Cooper HM, et al. The Netrin Receptor DCC Is Required in the Pubertal Organization of Mesocortical Dopamine Circuitry. The Journal of Neuroscience. 2011;31(23):8381-94.

6. Scattoni ML, Gandhy SU, Ricceri L, Crawley JN. Unusual repertoire of vocalizations in the BTBR T+tf/J mouse model of autism. PLoS One. 2008;3(8):e3067.

7. Barker GRI, Bird F, Alexander V, Warburton EC. Recognition Memory for Objects, Place, and Temporal Order: A Disconnection Analysis of the Role of the Medial Prefrontal Cortex and Perirhinal Cortex. The Journal of Neuroscience. 2007;27(11):2948-57.

8. Huang H, Michetti C, Busnelli M, Manago F, Sannino S, Scheggia D, et al. Chronic and acute intranasal oxytocin produce divergent social effects in mice. Neuropsychopharmacology : official publication of the American College of Neuropsychopharmacology. 2014;39(5):1102-14.
