# Supplementary material for: Adolescence is the starting point of sex-dichotomous COMT genetic effects
Source: Transl Psychiatry. 2017 May 30;7(5):e1141–. doi: 10.1038/tp.2017.109 (PMC5584523; doi:10.1038/tp.2017.109)
Supplement: Supplementary Information [file tp2017109x2.pdf]

	Pre-puberty			Post-puberty		
	Met	**Val**	**Tot.**	**Met**	**Val**	**Tot.**
N. Males	10	9	19	16	15	31
N. Females	12	8	20	20	19	39
Mean age	9.4±1.5 y.o.	8.9±1.7 y.o.	9.2±1.6 y.o.	19±4.8 y.o.	18.3±5 y.o.	18.7±4.9 y.o.
Right handed (%)	86%	88%	87%	78%	88%	83%
Psychiatric diagnosis	14 (9 females)	12 (7 females)	26 (16 females)	27 (14 females)	18 (11 females)	45 (25 females)
Medications	1 (female)	-	-	14 (7 females)	12 (7 females)	26 (14 females)
1.5T	10 (8 females)	11 (7 females)	21	9 (6 females)	5 (3 females)	14
3T	12 (4 females)	6 (1 female)	18	27 (14 females)	29 (16 females)	56

Supplementary Table 1. Demographic information in pre- and post-pubertal subjects from the Geneva cohort. These subjects were assessed in the MRI, IQ, Stroop, CPT, and digit span tests. 1.5T and 3T refer to the number of scans acquired at the 1.5T and the 3T machines divided by puberty, genotype and sex.

	Post-puberty		
	**Met**	**Val**	**Tot.**
N. Males	22	29	51
N. Females	15	17	32
Mean age	18±1,2 y.o.	17,6±1,1 y.o.	17.8±1,0 y.o.
Psychiatric diagnosis	23 (11 females)	31 (9 females)	54 (20 females)
Medications	3 (0 females)	7 (3 females)	10 (3 females)

Supplementary Table 2. Demographic information in post-pubertal subjects from the Roma cohort. These subjects were assessed in the WCST, IQ, Stroop, and digit span tests.

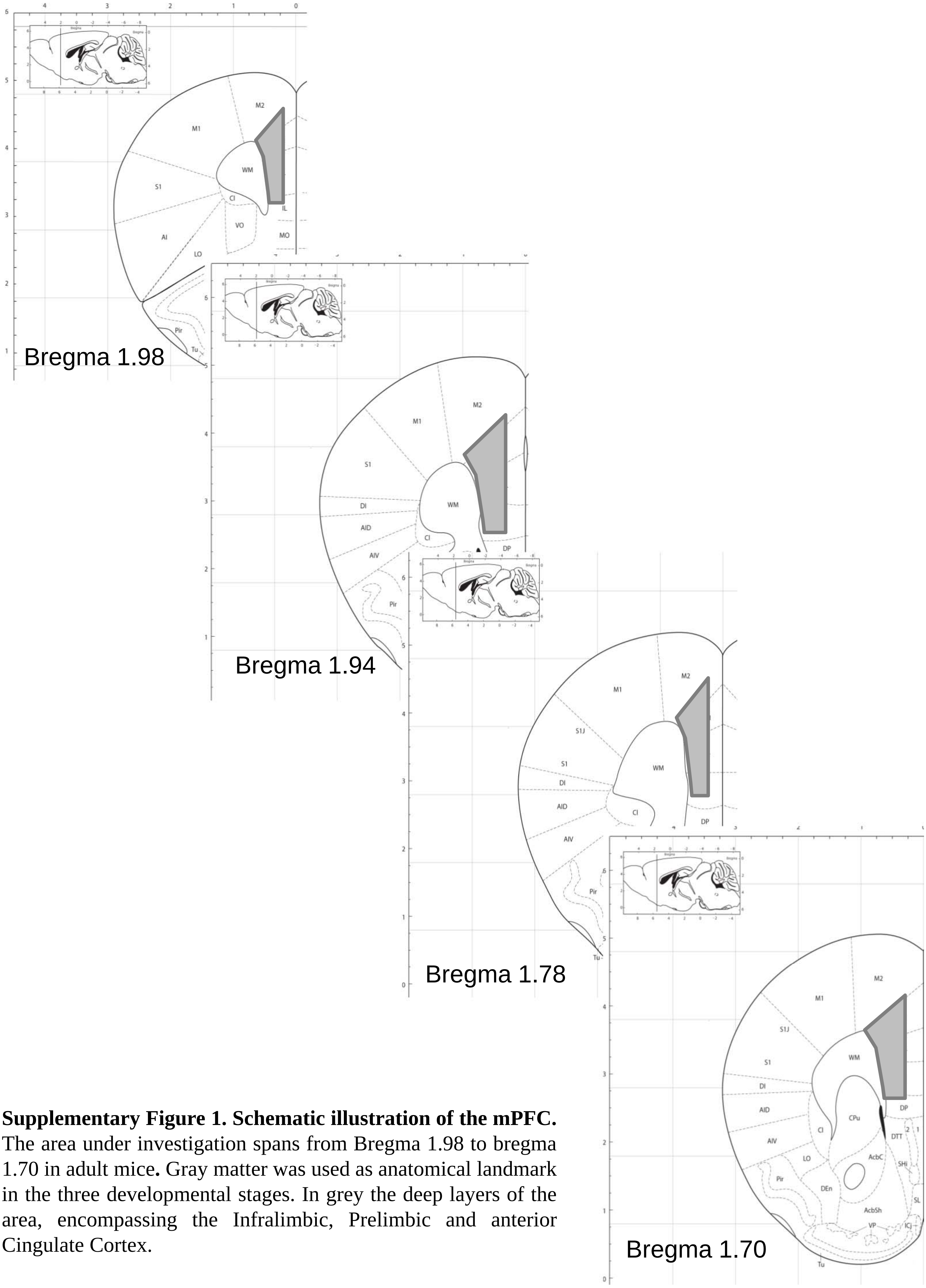

Supplementary Figure 1. Schematic illustration of the mPFC. The area under investigation spans from Bregma 1.98 to bregma 1.70 in adult mice**.** Gray matter was used as anatomical landmark in the three developmental stages. In grey the deep layers of the area, encompassing the Infralimbic, Prelimbic and anterior Cingulate Cortex.

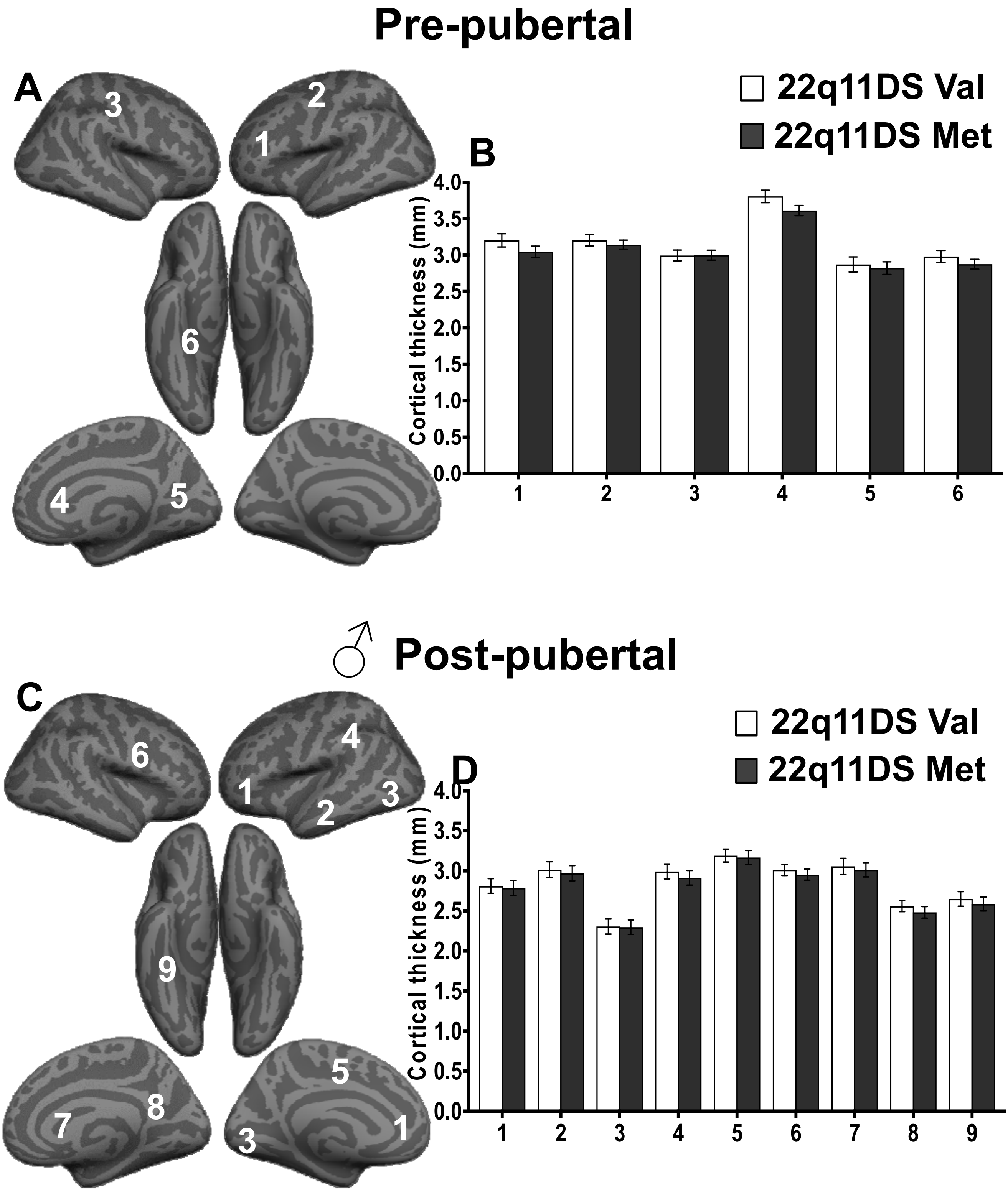

Supplementary Figure 2. (A) Cortical thickness do not significantly differ between 22q11DS COMT Met and Val carriers in pre-puberty. (**B**) Mean cortical thickness values for the clusters 1 to 6, that showed a significant difference between Met and Val carriers in post-puberty, are reported in the plots. (1=left precentral/inferior middle frontal cortices, 2=left superior frontal cortex, 3=right superior frontal cortex, 4=right medial superior frontal cortex, 5=precuneus, 6=posterior fusiform gyrus). (**C**) No significant differences were observed in male post-pubertal patients. (**D**) The plots represent mean cortical thickness values in male patients in the clusters where there was a significant difference between Met and Val post-pubertal females. (1=left superior frontal cortex, 2=left inferior frontal/insula/superior temporal cortices, 3=left occipital cortex, 4=left supramarginal gyrus, 5= left medial superior frontal cortex, 6=right inferior frontal cortex, 7=right medial superior frontal/anterior cingulate cortices, 8=right precuneus, 9=right posterior fusiform gyrus/lateral occipital cortex).*$p<0.05$ and **$p<0.01$ versus 22q11DS Val carriers.

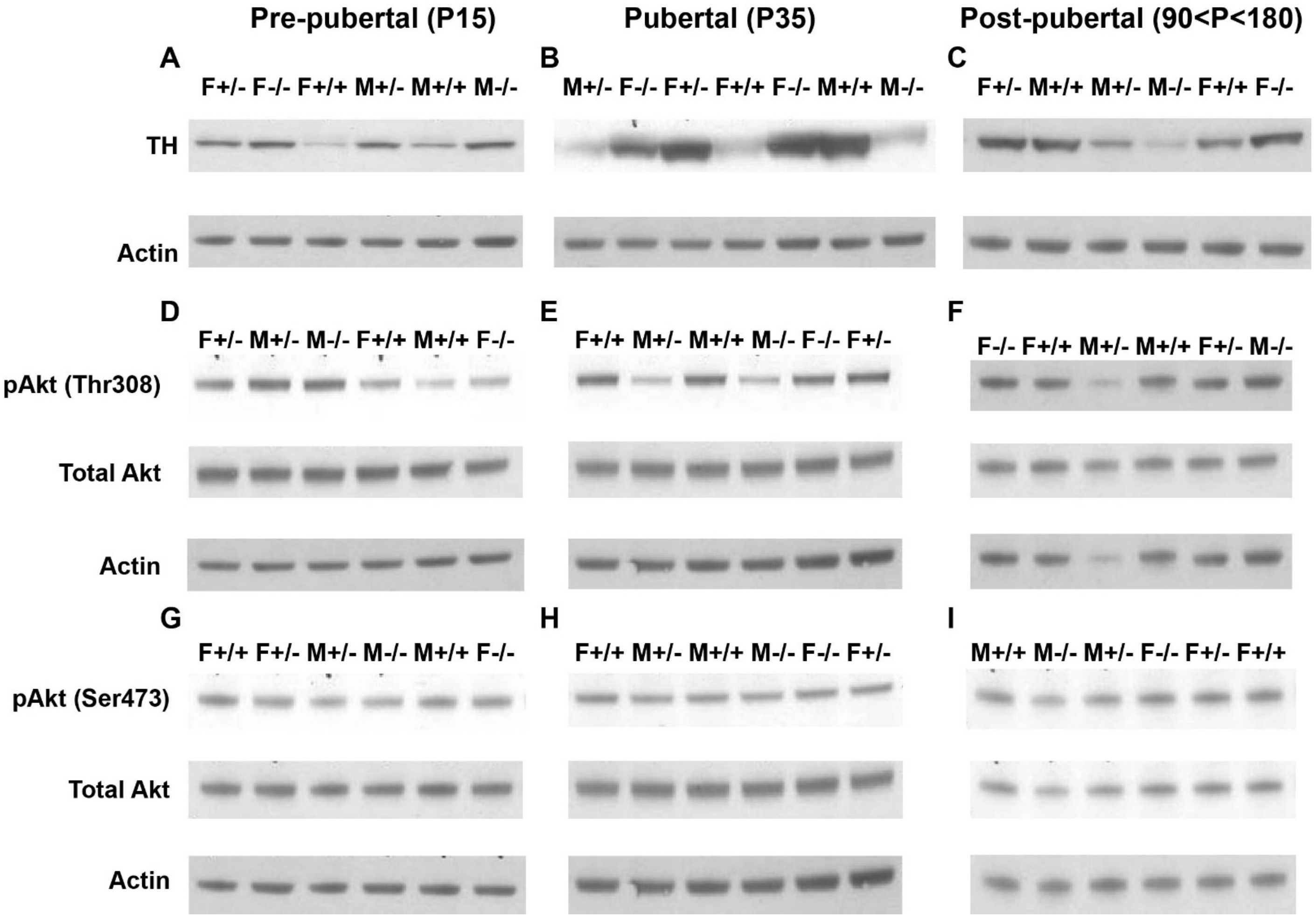

Supplementary Figure 3. Representative western Blot for the measurements of Tyrosine hydroxylase and Akt levels in the PFC. Representative western Blots showing **(A-C)** Tyrosine Hydroxylase (TH) and Actin, (**D-F)** pAkt(Thr308), Akt and Actin, **(G-H)** pAkt(Ser473), Akt and Actin levels from PFC extracts in both males (M) and females (F) for COMT wild-type (+/+), heterozygous (+/-) and null (-/-) knockout mice during three different perdiods of their development: **(A, D, G)** Pre-pubertal (P15), **(B, E, H)** pubertal (P35) and **(C, F, I)** Post-pubertal (90<P<180).

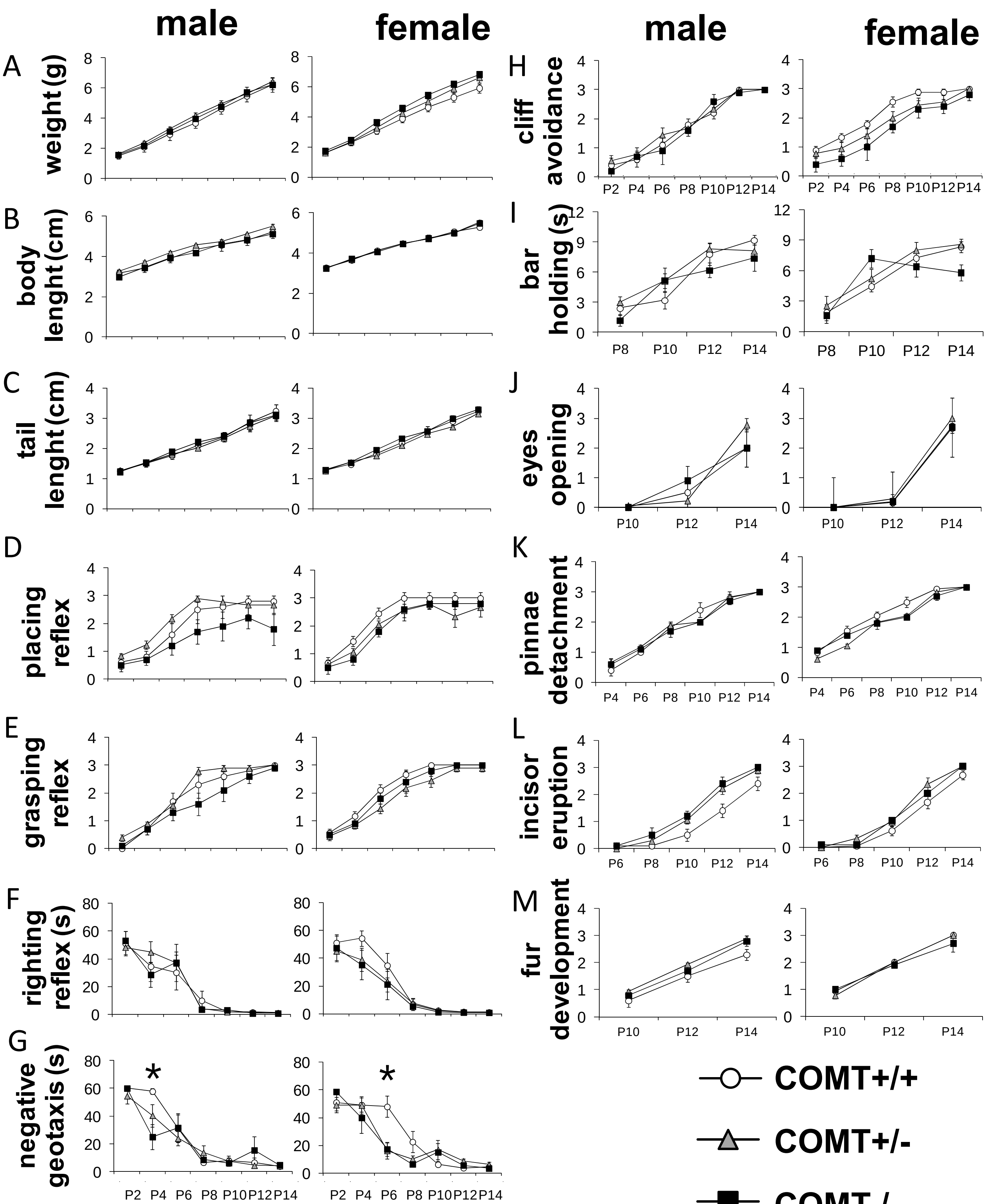

Supplementary Figure 4. COMT genetic reduction is not associated with delayed developmental milestones, it does not affect somatic growth and reflex development and it does not impair bar holding ability in pups and it in pups. COMT-/- and COMT+/- male and female mice are indistinguishable from their control littermates in the first two weeks of life for body weight (A), body length (B), tail length (C), placing reflex (D), grasping reflex (E), righting reflex (F). COMT-/- pups exhibit a developmental advantage in the negative geotaxis (G) at P4 in males and P6 in females. Cliff avoidance (H), bar holding (I), eyes opening (J), pinnae detachment (K), incisor eruption (L) and fur development (M) are not affected by lack of COMT gene. *p<0.05 vs COMT+/+.

	Degrees of freedom	F value	p value	Degrees of freedom	F value	p value	Degrees of freedom	F value	p value
**body weight**	12,216	1.2	0.3	6,216	0.3	0.9	12,216	0.9	0.5
**body length**	12,216	0.7	0.7	6,216	0.2	0.9	12,216	0.5	0.9
**tail length**	12,216	1.2	0.3	6,216	0.5	0.5	12,216	0.2	1
**forelimb placing reflex**	12,216	1.3	0.2	6,216	1.8	0.1	12,216	1.2	0.3
**forelimb grasping reflex**	12,216	1.4	0.2	6,216	0.7	0.6	12,216	1.5	0.1
**righting reflex**	12,216	0.4	1	6,216	1	0.4	12,216	0.7	0.7
**negative geotaxis**	12,216	2.6	0.002	6,216	0.9	0.5	12,216	1.9	0.03
**bar holding**	6,108	3.1	0.007	3,108	1.5	0.2	6,108	0.7	0.7
**cliff avoidance**	12,216	0.7	0.8	6,216	2.9	0.01	12,216	0.4	1
**fur development**	4,72	1.7	0.2	2,72	1.1	0.3	4,72	2.3	0.07
**eyes opening**	4,72	2.5	0.05	2,72	5.9	0.004	4,72	1.2	0.3
**pinnae detachment**	8,144	2.8	0.006	4,144	1.8	0.3	8,144	1	0.4
**incisor eruption**	8,144	3.4	0.001	4,144	0.8	0.5	8,144	0.7	0.7
**auditory startle**	2,36	1.1	0.3	1,36	4.3	0.04	2,36	0.3	0.8

Supplementary Table 3. Statistical analysis on pups' behavior observed form post-natal day 2 to post-natal day 14.

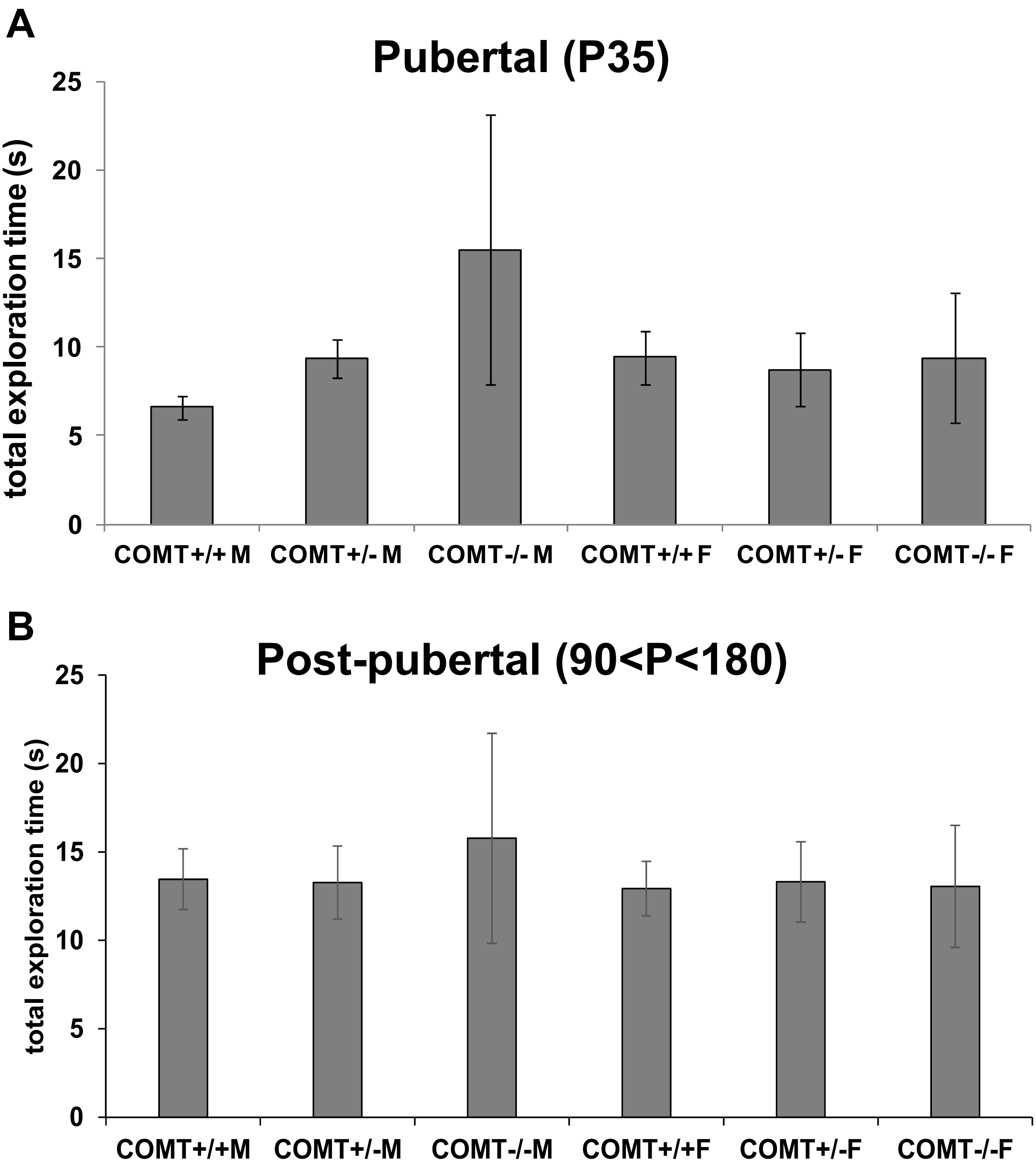

Supplementary Figure 5. Total objects exploration in the test phase. Adolescent (A) and adult (B) mice tested in the TOR task.

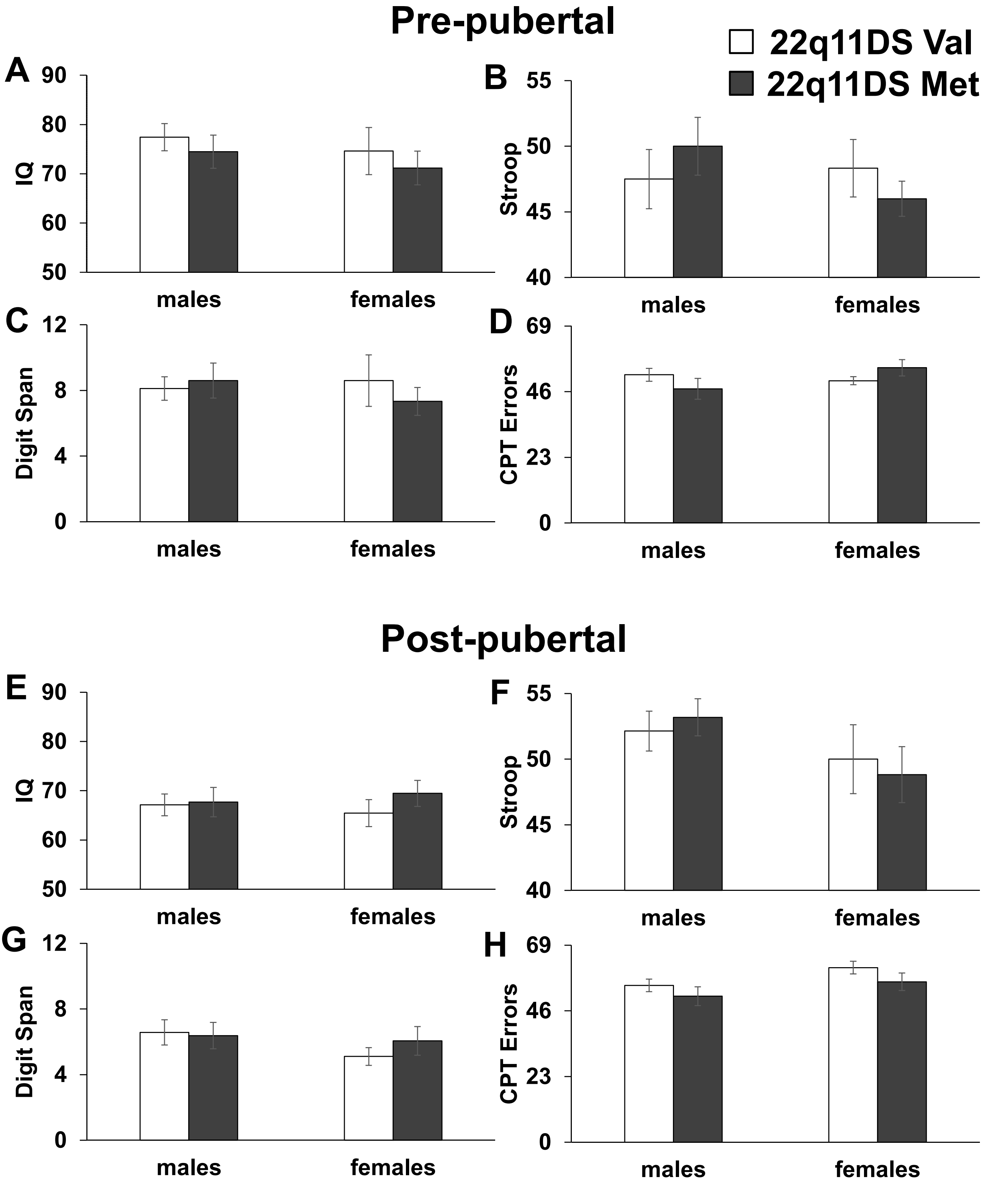

Supplementary Figure 6. Cognitive abilities showed by pre-pubertal (A-D) and post-pubertal (E-H) patients with 22q11DS assessed with IQ (A and E), interference index of the Stroop test (B and F), number of correct answers in the Digit span test (C and G), and commission errors in the CPT (D and H) sorted by their sex (males and females) and COMT ValMet genotype.
